# MET/HGF Co-Targeting in Pancreatic Cancer: A Tool to Provide Insight into the Tumor/Stroma Crosstalk

**DOI:** 10.3390/ijms19123920

**Published:** 2018-12-07

**Authors:** Chiara Modica, Dora Tortarolo, Paolo M. Comoglio, Cristina Basilico, Elisa Vigna

**Affiliations:** 1 Department of Oncology, University of Torino, 10060 Candiolo, Italy; chiara.modica@ircc.it (C.M.); dora.tortarolo@edu.unito.it (D.T.); elisa.vigna@ircc.it (E.V.); 2 Candiolo Cancer Institute, FPO-IRCCS, 10060 Candiolo, Italy; pcomoglio@gmail.com

**Keywords:** MET, HGF, metastasis, tumor microenvironment, pancreatic cancer, target therapy

## Abstract

The ‘onco-receptor’ MET (Hepatocyte Growth Factor Receptor) is involved in the activation of the invasive growth program that is essential during embryonic development and critical for wound healing and organ regeneration during adult life. When aberrantly activated, MET and its stroma-secreted ligand HGF (Hepatocyte Growth Factor) concur to tumor onset, progression, and metastasis in solid tumors, thus representing a relevant target for cancer precision medicine. In the vast majority of tumors, wild-type *MET* behaves as a ‘stress-response’ gene, and relies on ligand stimulation to sustain cancer cell ‘scattering’, invasion, and protection form apoptosis. Moreover, the MET/HGF axis is involved in the crosstalk between cancer cells and the surrounding microenvironment. Pancreatic cancer (namely, pancreatic ductal adenocarcinoma, PDAC) is an aggressive malignancy characterized by an abundant stromal compartment that is associated with early metastases and resistance to conventional and targeted therapies. Here, we discuss the role of the MET/HGF axis in tumor progression and dissemination considering as a model pancreatic cancer, and provide a proof of concept for the application of dual MET/HGF inhibition as an adjuvant therapy in pancreatic cancer patients.

## 1. The Hepatocyte Growth Factor Receptor (MET) and Its Ligand (HGF)

**Figure 1:**
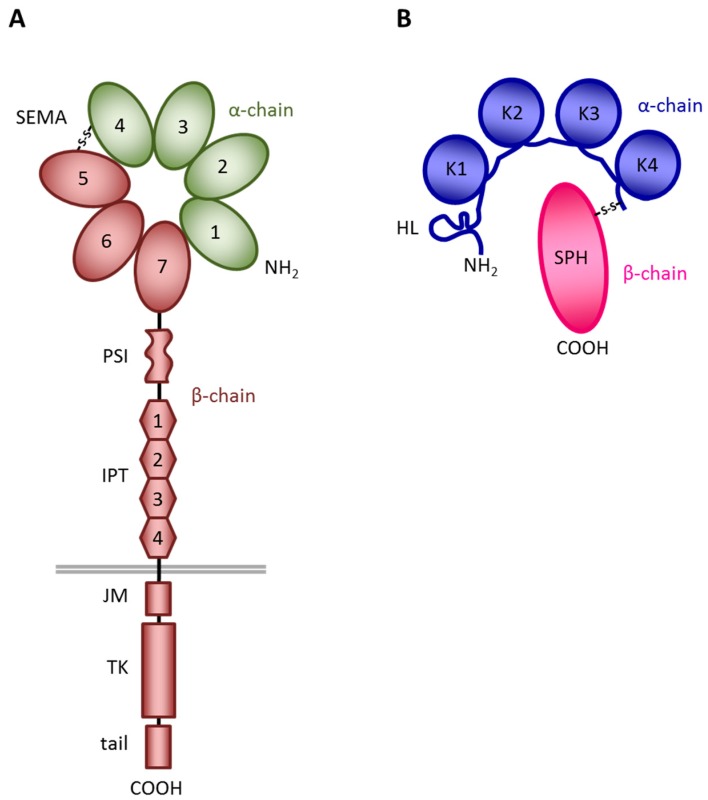
Schematic structure of MET and HGF. (**A**) MET structure. SEMA, a seven-bladed β-propeller domain shared with Semaphorins and Plexins; PSI, a cysteine-rich domain present also in Plexins, Semaphorins, and Integrins; IPT, four immunoglobulin-like domains present also in Plexins and Transcription factors; JM, juxtamembrane domain, a region containing residues that act as negative regulators of the kinase activity; TK, tyrosine kinase domain endowed with enzymatic activity; tail, a C-terminal domain containing the multifunctional docking site. In green the α-chain, in brown the β-chain of MET. The grey line represents the cell membrane. (**B**) HGF structure. HL, hairpin loop; K1–K4, kringle domains; SPH, serine-protease homology domain, devoid of protease activity. In blue the α-chain, in red the β-chain of HGF. S-S, disulphide bond linking the α- and β-chains of the two molecules.

MET was originally identified from a chemically transformed human osteosarcoma cell line as an oncogene resulting from the chromosomal rearrangement of the MET tyrosine kinase domain with a dimerization domain (TRP-MET) [1]. The *MET* gene was then mapped on chromosome 7q21-31 and its product identified as the tyrosine-kinase transmembrane protein, which is the high-affinity receptor for the Hepatocyte Growth Factor (HGF) [2,3]. MET is a heterodimeric protein made of two disulphide-linked chains of 50 kDa (α) and 145 kDa (β) [4]. The extracellular portion of MET has a modular structure. It contains: (i) an N-terminal SEMA domain (found also in semaphorins and plexins [5]) composed of 500 residues and characterized by a seven-bladed β-propeller structure [6]; (ii) a PSI domain (present also in Plexins, Semaphorins, and Integrins), comprising about 50 residues and containing four disulfide bonds; and (iii) four IPT domains (Immunoglobulin-like fold present also in Plexins and Transcription factors) [7,8]. The intracellular portion of MET includes: (i) a juxtamembrane region, which acts as negative regulator of the kinase activity [9,10]; (ii) a tyrosine kinase domain, which includes the major phosphorylation site that, following receptor dimerization and transphosphorylation, triggers the signaling cascade; and (iii) a C-terminal tail, which acts as multifunctional docking sites for the recruitment and activation of several intracellular signal transducers and adaptors [11,12] (Figure 1A).

HGF was identified by two independent studies as a mitogen for hepatocytes [13,14,15] and as a fibroblast-secreted scatter factor (SF) able to induce dissociation and motility of epithelial cells [16]. In 1991, HGF and SF were recognized as the same protein responsible for the induction of a variety of biological responses through the binding to MET [2,17]. HGF (Figure 1B) is a member of the plasminogen-related growth factor family, and it is synthesized as an inactive single-chain precursor (pro-HGF), which is proteolytically processed in the extracellular environment to generate the biologically active molecule [13,18]. The resulting mature HGF is a 90 kDa heterodimer consisting of two chains of 69 kDa (α) and 34 kDa (β), respectively, linked by a disulphide bridge. The α subunit includes an N-terminal hairpin loop and four kringle domains; the β subunit consists of a serine protease homology domain lacking enzymatic activity [19]. The HGF–MET interaction is highly complex, and engages different sites both on the ligand and the receptor. The α-chain of HGF binds MET at high affinity, interacting with both the IPT [20] and the SEMA [21,22] domains of the receptor. Conversely, the HGF β-chain binds the MET SEMA domain at low affinity [6,23]. Importantly, the cooperation between the two chains is necessary for HGF biological activity; while the α-chain is sufficient for MET binding, the β-chain is required for MET activation [24]. In particular, conformational changes occurring in the β-chain of HGF upon proteolytic cleavage allow for allosteric stabilization of the MET/HGF interaction and are critical for activity [25] (Figure 1B).

## 2. MET/HGF Signaling in the Physiological Context

**Figure 2:**
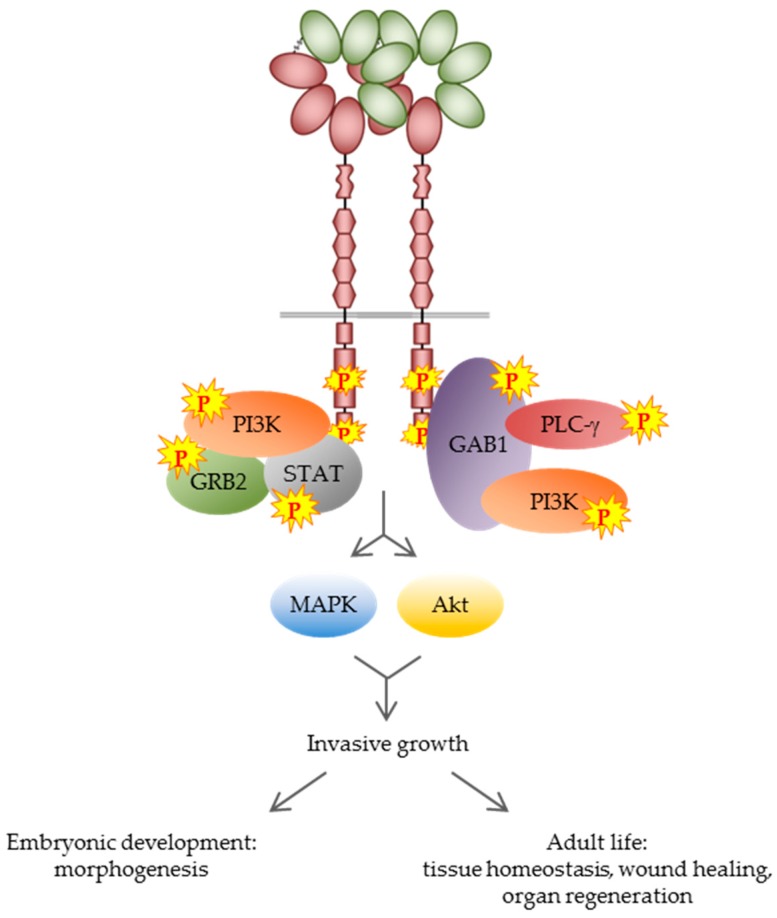
MET signaling in the physiological context. Upon MET activation, signal-relay molecules are recruited to the receptor, resulting in activation of the MAPK (mitogen-activated protein kinase)/Akt signaling pathways. This process controls the genetic program known as ‘invasive growth’ that in the physiological context plays a pivotal role in embryogenesis and in tissue homeostasis during adult life. PI3K, phosphoinositide 3-kinase; GRB2, growth factor receptor-bound protein 2; STAT, signal transducer and activator of transcription protein; GAB1, GRB2-associated-binding protein 1; PLC-γ, phospholipase Cγ1

The interaction between HGF and MET induces receptor dimerization, which results in *trans*-phosphorylation of the two tyrosine residues Y^1234^ and Y^1235^ within the catalytic domain [26], and the subsequent phosphorylation of tyrosines Y^1349^ and Y^1356^ within the C-terminal docking site, allowing for the recruitment of signal-relay molecules [11]. MET activation spurs a complex network of unconventional and pleiotropic intracellular signals that control a multifaceted genetic program, known as ‘invasive growth’ [27], that promotes cell proliferation, scattering, motogenesis, and survival. In epithelial cells, MET activation is followed by the acquisition of polarity and tubule formation [28,29]. This program is pivotal during embryonic development [30] and plays an important role in adult life, regulating tissue homeostasis, wound healing, and organ regeneration [31,32,33,34] (Figure 2).

In the early phases of mouse embryogenesis, HGF and its receptor MET are co-expressed in endodermal and mesodermal structures along the rostro-caudal axis [35]. However, during organogenesis, autocrine signaling stops in favor of a paracrine mode of activation, in which MET, expressed by epithelial and myoblast progenitors, is stimulated by the HGF produced by mesenchymal cells [36]. This paracrine loop is able to conjugate proliferative and anti-apoptotic stimuli with a strong motogenic activity into a single ‘invasive growth’ program that is necessary for embryonic development. In the adult, the signaling program activated by MET and HGF during organogenesis is exploited in the presence of wounds and tissue damage. In this context, pro-inflammatory cytokines, such as interleukin-1 (IL-1), interleukin-6 (IL-6) and tumor necrosis factor-α (TNFα), induce HGF and MET overexpression, while the stroma contributes to HGF activation by upregulating the transcription of proteases involved in proHGF processing [37,38]. HGF induces a re-organization of the focal adhesion components, actin stress fibers, and microtubules by eliciting the recruitment of the intracellular effectors GAB1, Akt, ERK (extracellular signal-regulated kinase 1), and RhoA (Ras homolog gene family member A), and it promotes the proliferation and migration of epithelial cells at wound edges [39]. In the liver and kidney, persistent damage leads to the deposition of extracellular matrix by myofibroblasts (fibrosis), sustained by TGF-β (transforming growth factor-β) upregulation [40]. This process is antagonized by HGF-mediated MET activation, which leads to downregulation of TGF-β transcription and Smad inhibition through ERK [41,42]. Moreover, HGF sustains the proliferation of renal epithelial cells by activating the Ras-dependent MAPK pathway and the PI3K-AKT pathways, and protects them from apoptosis by upregulating anti-apoptotic effectors, such as BCL-2 (B-cell lymphoma-2) [43]. In vivo, liver damage leads to a sharp increase in HGF production, which provides mitogenic and anti-apoptotic stimuli for organ repair [44]. Accordingly, organ reconstitution after hepatectomy is abrogated in MET conditional knock-out mice [34]. Finally, the MET/HGF pathway plays a relevant role in cardiovascular protection in response to both acute and chronic damage [45]. After ischemic injury, for example, MET activation provides strong anti-apoptotic stimuli for cardiomyocytes through the PI3K/AKT and MAPK pathways [46].

## 3. MET/HGF Signaling in Cancer

Unfortunately, the same highly regulated events that mediate MET/HGF biological responses during embryo development and contribute to maintain tissue homeostasis in the adult are unleashed in cancer. The first evidence of a link between MET and human oncogenesis was obtained from the observation of a number of germline MET mutations in patients with hereditary papillary renal cell carcinomas (HPRCC) [47]. Since then, altered MET functions were identified as a hallmark of several cancer types, including carcinomas, sarcomas, and brain tumors, and may occur through gene amplification, deletions, mutations, receptor overexpression, or disruption of normal paracrine signaling [48,49,50].

In a limited number of tumors, *MET* genetic lesions—either mutations [47] or amplifications [51]—lead to the constitutive activation of MET. Oncogenic mutations are concentrated in domains critical for ligand binding or receptor signaling. In the first case, mutations occur in the extracellular SEMA domain, likely affecting the ligand–receptor interaction or the ability of MET to dimerize, and have been detected in lung, gastric, and breast cancer [48], as well as in cancers of unknown primary (CUPs) [52]. In the second case, point mutations or deletions affect the juxtamembrane domain of MET—inducing sustained signaling by attenuating receptor ubiquitination and degradation—or the catalytic domain of the receptor, causing MET hyper-activation [49,53]. Both cases result in prolonged and uncontrolled receptor signaling, which generates a cell autonomous mechanism mostly independent of ligand binding. Similar aberrant activation may derive from gene amplification. Such *MET* genetic lesions act as drivers of malignant transformation, and confer a growth advantage to tumor cells [54]. In this context, cancer cells depend on MET signaling for their growth and survival, a phenomenon that is common to other tyrosine kinase receptors and that has been indicated as ‘oncogene addiction’ [55]. As a consequence, blockade of the MET pathway results in inhibition of tumor growth.

The generation of a MET/HGF autocrine loop by anomalous co-expression of ligand and receptor in the same cell is another way by which cancer cells may achieve continuous MET activation. This mechanism has been described mainly in non-epithelial human tumors, such as osteosarcomas [56], glioblastomas [57], and multiple myelomas [58]. Tumors of mesenchymal origin, such as osteosarcomas and rhabdomyosarcomas that physiologically express HGF, can acquire an aberrant expression of MET leading to the establishment of an autocrine loop that sustains constitutive activation of the signaling pathway [56]. Similarly, tumors deriving from cells that physiologically express MET, including glioblastomas and breast carcinomas, can acquire anomalous HGF expression [59,60]. The presence of autocrine loops is indicative of aggressive cancers with a poor prognosis [59].

However, in the vast majority of tumors, aberrant MET signaling derives from the upregulation of wild-type MET or HGF transcription, leading to receptor and/or ligand overexpression [61,62], or from extensive proteolytic conversion of pro-HGF stored in the extracellular matrix, resulting in unrestrained availability of biologically active HGF. In such conditions, the HGF secreted by the reactive tumor stroma acts on cancer cells expressing MET to promote pro-invasive and anti-apoptotic responses. MET or HGF overexpression frequently arises as a consequence of transcriptional upregulation in response to adverse environmental cues, such as inflammation and hypoxia. In fact, both *MET* and *HGF* gene promoters contain binding sites for transcription factors, including hypoxia-inducible factor 1α (HIF1α) and nuclear factor-κB (NF-κB), that are induced or activated by stress conditions [50]. Differently from the context of ‘oncogene addiction’—where aberrant receptor signaling originates from a genetic lesion and drives cancer initiation—here, wild-type *MET* activation represents a strategy harnessed by otherwise transformed tumor cells to overcome barriers to their progression and to boost their malignant phenotype. This condition is defined as ‘oncogene expedience’ [63]: cancer cells exploit the invasive growth program elicited by MET to increase their survival, motility, and invasiveness. In line with this assumption, wild-type MET overexpression is frequently associated with highly aggressive tumors, in which ‘pioneer’ cells exploit the invasive growth program elicited by MET to detach from the primary site, invade distant tissues, and establish metastatic lesions [27]. In light of these considerations, therapies targeting the MET/HGF pathway in a context of expedience may impair the survival and the ability to disseminate of cancer cells, but are not expected to reduce tumor growth.

## 4. MET/HGF Signaling in the Crosstalk between Cancer Cells and the Tumor Microenvironment

**Figure 3:**
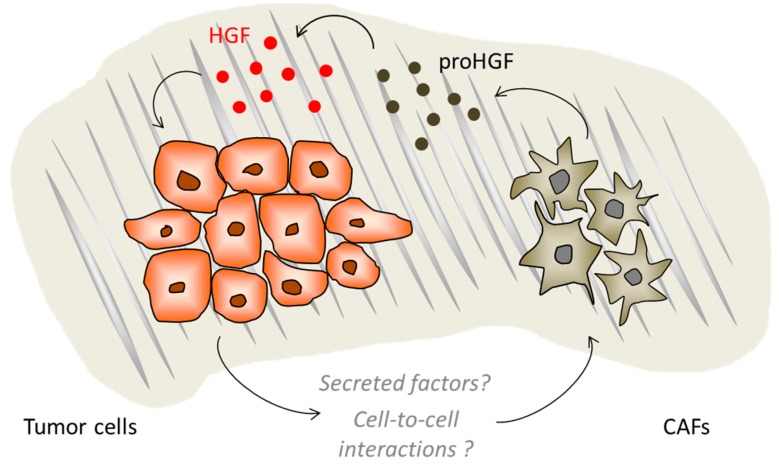
The dynamic bidirectional tumor/stroma interaction. Cancer-associated fibroblasts (CAFs) secrete pro-HGF that is converted in the active mature form by enzymes acting in the tumor microenvironment. Tumor cells can influence CAF activity by mechanisms that have not yet been completely elucidated. The fibrotic components of the extracellular matrix are depicted in grey.

The contribution of the tumor microenvironment (TME) to cancer progression is becoming increasingly relevant. It is now well-established that the malignant phenotype does not develop in a strictly cell-autonomous way, but through a complex interplay that takes place between the tumor cells and the surrounding stroma [64]. Tumors are complex tissues where cancer cells coexist with several non-malignant cells that produce a rich extracellular matrix (ECM) and a variety of molecular effectors. The cellular fraction of TME is composed by a multitude of stromal cell types, including fibroblasts, pericytes, endothelial and mesenchymal cells, as well as cells of the immune system [64]. Non-malignant stromal cells are recruited and corrupted by cancer cells to produce cytokines, chemokines, growth factors, and matrix remodeling enzymes, resulting in a complex and dynamic network that often retains a tumor-promoting activity at all stages of carcinogenesis [65]. Cancer-associated fibroblasts (CAFs) are often referred to as myofibroblasts due to the acquisition of a multi-spindled shape and positivity for α-smooth-muscle actin (α-SMA). CAFs produce higher levels of tumor-promoting factors, such as HGF, FGF, TGFβ, PDGF, VEGF, and EGF, as compared to normal fibroblast; these factors act in a paracrine manner on the cognate receptors expressed on adjacent tumor cells [66,67,68]. In addition, CAFs secrete abnormal ECM components, such as tenascin, periostin, SPARC, and collagen, which overall contribute to a more rigid and contractile ECM that favors tumor growth and survival [69]. In fact, TME-associated ECM is different from that of normal tissues: in particular, elevated collagen crosslinking causes alterations of its biochemical properties, increasing tissue rigidity. The ECM is composed of a variety of macromolecules with distinctive properties, including proteins, glycoproteins, proteoglycans, and polysaccharides, and represents a dynamic scaffold for the growth and invasion of tumor cells [70,71]. Moreover, the activity of matrix metalloproteases (MMPs) contributes to ECM remodeling, inducing the release of chemokines, growth factors, and pro-angiogenic factors [72,73]. Altogether, the activated stroma creates a dense fibrosis called ‘desmoplastic reaction’ around the tumor, which constitutes a physical obstacle for drug penetration and affects tumor vascularization [74]. Among the plethora of factors secreted in the tumor microenvironment, HGF plays a relevant pro-malignant role, triggering cancer cell invasion and metastatic dissemination via paracrine stimulation of its receptor on tumor cells. MET activation further stimulates HGF production by CAFs, thus establishing a dynamic bidirectional ‘tumor-host signaling program’ [75] (Figure 3).

The malignant behavior of cancer cells is nurtured by the collateral pro-angiogenic activity of the MET/HGF axis in endothelial (ECs) and stromal cells [76]. MET upregulation in ECs is required to promote the early steps of angiogenesis, in which ECs change shape, invade the extracellular matrix, and proliferate [77]. Furthermore, HGF stimulates angiogenesis both directly—by binding to its receptor on the surface of endothelial cells—and indirectly—by prompting non-EC populations to produce pro-angiogenic factors, such as vascular endothelial growth factor (VEGF)-A [78]. Overall, MET and HGF concur to increase neovascularization, tumor expansion, and the release of neoplastic emboli into the bloodstream, regardless of the state of MET activation in cancer cells.

Furthermore, MET pathway activation also influences macrophage polarization, inducing a shift of their phenotype from an immunological active phenotype (M1) to a trophic, growth-stimulating state (M2), promoting an immunosuppressive environment [50,79].

In order to allow for the execution of the multifaceted metastatic process, a favorable microenvironment around the tumor tissue is mandatory, as well as plasticity and migration of cancer cells. Metastases arise from a selected cell subpopulation that resides in the heterogeneous primary tumor [80]. The acquisition of a metastatic phenotype requires the aberrant execution of a biological plan, including the activation of the invasive growth program, in which cancer cells acquire the ability to disrupt cell-to-cell interactions, migrate through the extracellular matrix, spread through lymphatic or hematopoietic vessels, and finally extravasate, survive, and proliferate in tissues other than their site of origin [61]. The MET/HGF pathway acts as a key player at multiple stages during the development of the metastatic disease, from cellular dissociation within the primary tumor to cellular re-association within the metastatic niche [27]. For instance, HGF triggers the destabilization of cell–cell adherents junctions, shapes cytoskeleton reorganization, modulates integrin functions, and it stimulates the MMP-mediated proteolysis of ECM [27,50]. Recently, the invasive growth concept has been redefined to incorporate the process of epithelial-to-mesenchymal transition (EMT) [50]. EMT is a reversible, plastic condition through which cancer cells undergo a global change in cell architecture and phenotype: this confers the ability to spread out from the native tissue and to establish tumor colonies in distant organs [81]. EMT displays a number of analogies with physiological processes, such as re-epithelization during wound healing and cell migration during embryonic development [82]. The role of MET in organogenesis and tubule formation has been thoroughly studied and well-described: after HGF stimulation, cells form protruding branches and undergo a partial EMT, which results in invasive, spindle-shaped cells that form long cytoplasmic extensions in the surrounding matrix [83]. The cells within the extensions must resist anoikis to divide and arrange in rows. Ultimately, cells re-differentiate to the epithelial phenotype by restoring polarity, and subsequently the tubules evolve into solid cords with a continuous lumen [83]. The same MET/HGF-dependent mechanism described in a physiological context is exploited by cancer cells to disseminate from the tissue of origin and to colonize a different organ [84]. Migrating cancer cells seem to evade anoikis by MET-dependent upregulation of the cytoskeleton adhesion receptors, which allows them to interact with the new surrounding cells and to elude apoptosis [85].

## 5. MET/HGF Signaling in Pancreatic Cancer

Since the pioneer studies published in 1995 [86], the MET/HGF system has gained growing attention with respect to its role in the pathogenesis of pancreatic cancer (for a review see [87]). About 94% of these cancers develop from the exocrine tissues and are classified in four subtypes: ductal adenocarcinoma, acinar cell carcinoma, intraductal papillary-mucinous neoplasm, and mucinous cystic neoplasm [88]. Pancreatic Ductal Adenocarcinoma (PDAC) accounts for the majority of cases, representing an aggressive malignant disease with a 5-year survival rate lower than 5% [89]. Multifactorial reasons are responsible for this extremely morbid outcome, such as diagnosis at late stage, rapid metastatic progression, and resistance to conventional therapy. PDAC is characterized by an intense stromal desmoplastic reaction [90] that accounts for about 80% of the cancer nodule [91]. This fibrotic stroma is produced mainly by a special type of CAFs, the activated pancreatic stellate cells (PSCs), which are entwined into the acinar cells of the pancreas [90]. In tumorigenic conditions, a prolonged activation of PSCs and the extensive crosstalk with cancer cells perpetuate the desmoplastic reaction, leading to the deposition of excessive amounts of ECM proteins around malignant cells. The fibrous layer may restrict blood flow, oxygen availability, and the delivery of chemotherapeutic agents, inducing, respectively, metabolic switches, tissue hypoxia, and drug resistance. Additionally, the dense stromal compartment also represents a reservoir of cytokines and growth factors. An example is given by HGF, which is secreted, accumulated, and activated within the stromal tissue surrounding the malignant cells [92]. High levels of stromal HGF have been associated with worsened overall survival of pancreatic cancer patients [92]. Interestingly, while activating *KRAS* mutations followed by mutation of *p16* and loss of p53 and SMAD4 are often considered as initiating events in pancreatic cancers [93], dysregulation of MET activity correlates with the development of an aggressive phenotype [94]. Compared to the normal tissues, MET protein displays on average a sevenfold increase of expression in pancreatic tumor lesions; this feature has been recently linked to the activity of DYRK1A, a kinase involved in the regulation of the MET turn-over [95]. MET overexpression is directly correlated to tumor grade [94,96,97], and likely supports the pro-metastatic role of TME by sensitizing cancer cells to HGF-driven signaling [98].

Interestingly, PSCs seem to be capable of travelling within the body together with cancer cells to colonize distant organs [99]: in patients with late-stage pancreatic cancer, CAFs were identified within tissues containing pre-metastatic or metastatic lesions [100]. Moreover, at the metastatic site, CAFs were found to be involved in the accumulation of a desmoplastic stroma similar to that of primary tumors [100]. These data suggest that heterogeneous stromal signaling is required also at the metastatic site, and support an involvement of the MET/HGF paracrine loop in the metastatic spreading of PDAC tumors [101,102]. The MET/HGF axis has been proven to further stimulate HGF production by mesenchymal cells, with the formation of a feed-forward loop [103]. Furthermore, urokinase plasminogen activator (uPA), one of the factors promoting HGF maturation from an inactive precursor to the active form [18], was found itself to be increased by active HGF, overall resulting in a continuous positive feedback loop amplifying both HGF and uPA [104].

The MET/HGF pathway is involved in the complex crosstalk between tumor and stroma, in particular in the interaction between cancer cells and activated PSCs [105]. In this regard, targeting HGF by an antibody (AMG-102) induces stromal reprogramming resulting in a reduction of collagen deposition [106]. This result suggests that the paracrine signaling between stroma and cancer through the MET/HGF axis is not unidirectional towards the activation of the tumor, but also favors the activation of the stromal compartment itself, through a still unknown process that deserves further investigation.

Although tumor resection remains the keystone of treatment for pancreatic cancer, only a small fraction of patients are candidates for surgery at the time of diagnosis [107], as early systemic dissemination is one of the features characterizing the disease. Currently, the treatment of choice for advanced and metastatic pancreatic cancer is chemotherapy through gemcitabine administration, often in combination with nab-paclitaxel. Unfortunately, most patients develop resistance after a few weeks of treatment [108], caused either by intrinsic genetic factors (primary resistance) or by acquired mechanisms. Among the latter, a role can be envisaged for de novo aberrant activation of signaling pathways regulating nodal tumor–stroma interplays [108]. MET aberrant activation in pancreatic cancer is involved in the onset of resistance to gemcitabine by different mechanisms. On one side, MET has been identified as a marker of pancreatic cancer stem cells (CSCs) [109], and on the other the MET/HGF axis supports the mesenchymal network [110]. Preclinical studies demonstrated that gemcitabine-resistant PDAC cells express high levels of stem cell markers, indicating that the drug treatment selects a subpopulation of tumor cells with stem-like features. In several solid tumors, MET is expressed in stem/progenitor cells, fostering pro-survival and anti-apoptotic signals (MET ‘inherence’) [50]. In line with these observations, in vitro studies demonstrated that targeting the MET pathway specifically interfered with the pancreatic CSC subpopulation, inducing apoptosis and reducing migration of resistant cells, and synergistically interacted with anti-tumor gemcitabine activity [111,112]. Furthermore, gemcitabine-resistant cancer cells acquire the hallmarks of EMT in association with an increment of MET phosphorylation and the expression of the stem cell marker CD (cluster of differentiation) [113]. In vivo, knock-out of the *MET* gene in a mouse model of spontaneous pancreatic neoplasia resulted in a reduction of neoplastic cell proliferation, a predictive marker for chemotherapeutic responses and clinical prognosis [114]. Finally, the inhibition of the tumor–stroma interaction by MET/HGF interception induces stroma remodeling, which results in increased drug delivery and restoration of the response to gemcitabine [106,115].

## 6. Concomitant Inhibition of MET and HGF: A Strategy to Interrupt the Dialogue between Tumor and Stroma

Since the tumor microenvironment significantly contributes to cancer progression and metastasis, and since the malignant phenotype of cancer cells may be exacerbated by the presence of overexpressed/overactivated wild-type MET in dialogue with stromal-derived HGF, the development of a strategy aimed at disrupting this crosstalk by concomitantly targeting receptor and ligand can be envisaged. MET and HGF co-targeting has been previously investigated in MET-addicted tumors and in cancers expressing a MET/HGF autocrine loop. In these contexts, the combination treatment improves the efficacy of each monotherapy [116,117]. In a recently published work [118], we proposed an approach for dual MET/HGF inhibition by co-administration of MvDN30, a MET-neutralizing antibody, and decoyMET^K842E^, a soluble MET receptor endowed with HGF-sequestering ability. MvDN30 is a chimeric Fab fragment that acts as a potent MET inhibitor by promoting the physical removal of the MET receptor from the cell surface, thus shutting off MET signaling [119]. Upon binding to MET, MvDN30 enhances the activity of receptor shedding that is mediated by the surface ADAM10 protease, supposedly by inducing conformational changes in the receptor that reveal a specific motif recognized by the metalloprotease. As a result, the number of MET receptors exposed on the cell surface is strongly reduced, and soluble extracellular domains are released: they retain functional ligand binding sites and therefore sequester the environmental HGF, preventing binding and activation of residual receptors. To potentiate this sponge-like HGF-neutralizing activity, we co-administered the antibody with a recombinant decoyMET, engineered by truncation of the MET receptor immediately upstream of the transmembrane domain [120] and by insertion of a specific amino acid substitution at position 842, where a lysine residue has been converted into a glutamic acid (decoyMET^K842E^). This sequence modification, which does not affect the affinity for HGF, is sufficient to completely abrogate the interaction between decoyMET and the antibody, thus preventing the reciprocal neutralization of either component.

**Figure 4:**
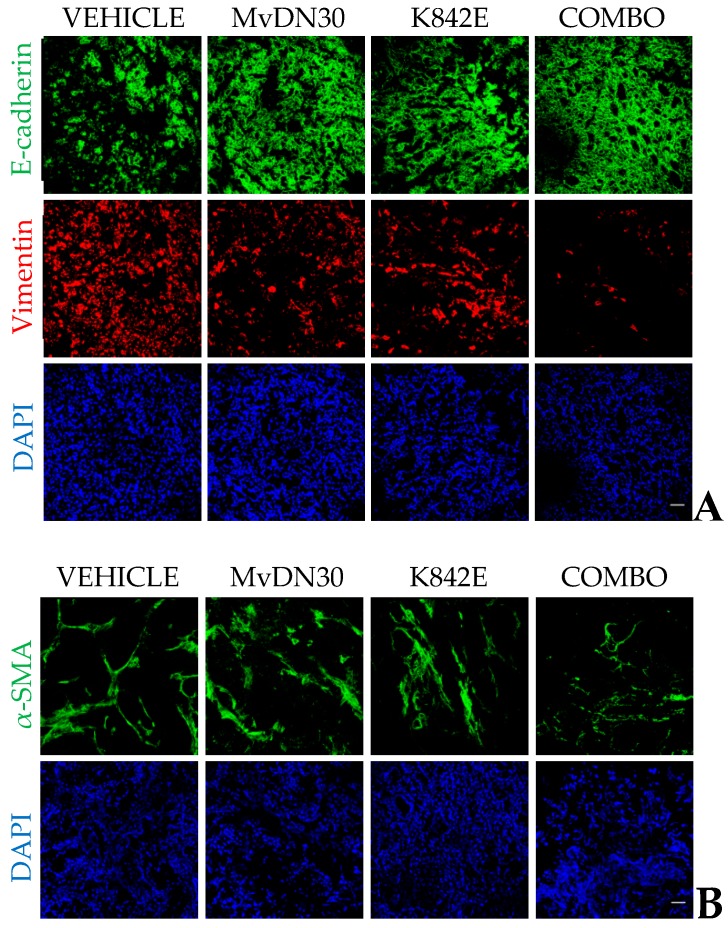
Concomitant MET/HGF targeting induces a reversion of the epithelial-to-mesenchymal transition (EMT) phenotype as well as a decrease of activated α-SMA positive fibroblasts in an orthotopic model of pancreatic cancer. HPAF-II cells were orthotopically injected in SCID mice genetically modified to secrete physiological levels of human HGF (hHGF knock-in); after 2 days, mice were divided into four groups and treated with vehicle, MvDN30 (10 mg/kg), decoyMET^K842E^ (10 mg/kg), or the combination of the two in a 1:1 ratio. Treatments were administered every 2 days by intraperitoneal injection. After 5 weeks of treatment, mice were sacrificed, and primary tumors were collected and processed for an immunofluorescence analysis. (**A**) E-cadherin and vimentin staining. Bar is 50 µm. (**B**) α-SMA staining. Bar is 50 µm.

Concomitant MET/HGF targeting by the dual antibody/decoy strategy proved to be extremely effective in blocking HGF-driven MET activation both in vitro and in vivo. Remarkable results were obtained in a mouse model of pancreatic adenocarcinoma (PDAC) built by orthotopic injection of human PDAC cells—expressing wild-type MET—in SCID (severe combined immunodeficient) mice genetically modified to secrete physiological levels of human HGF (hHGF knock-in) that bypass the problem of ligand/receptor species-specificity. In this model, the combination of MvDN30 and decoyMET^K842E^ targeted directly human cancer cells, strongly impairing MET phosphorylation in primary tumors, and affecting the stromal component of primary tumors. In fact, the combo treatment induced a reversion of the EMT phenotype (measured as vimentin/E-cadherin ratio) as well as a decrease of activated α-SMA positive fibroblasts (Figure 4). In line with these observations, which point to the attenuation of the invasive phenotype of the primary tumors, the dissemination of pancreatic cancer cells was dramatically reduced. These data support a pivotal role for the MET/HGF axis in EMT and stromal reprogramming [50], and suggest that concomitant receptor and ligand targeting might be beneficial to impair tumor cells spreading in highly metastatic cancers, such as PDAC.

Consistent with our results, a ‘triple therapy’ (inhibition of both HGF and MET combined with gemcitabine) has been recently shown to achieve stronger inhibition of both primary tumor growth and metastatic dissemination compared to the single treatments or dual combinations in a mouse model of pancreatic cancer [110].

## 7. Conclusions

Activation of the MET/HGF pathway is at the same time a catalyst and a consequence of the pro-tumoral microenvironment. TME represents a significant source of HGF, which may trigger signaling cascades in cancer cells, thus fostering their survival and dissemination. Conversely, MET/HGF signaling influences TME by acting directly on stromal MET-expressing cells, either resident or attracted from blood circulation.

The results obtained with the combination of MvDN30 and decoyMET^K842E^ suggest that the dual decoy/antibody strategy on one side may revert the EMT process of cancer cells, and on the other may act on the stromal component by reducing CAFs activation and ECM deposition. These results support the hypothesis of a bidirectional signaling between stroma and cancer through the MET/HGF axis, indicating that tumor cells might prompt the activation of the stromal compartment.

Moreover, the MET/HGF co-targeting may represent an option as an adjuvant therapy in pancreatic cancer. The standard treatment for advanced pancreatic cancer is chemotherapy, namely Gemcitabine alone or in combination with Nab-paclitaxel. However, often patients undergo resistance to chemotherapy. One of the causes of PDAC chemoresistance relies on the presence of an extensive desmoplastic reaction that surrounds the tumor, creating a physical barrier to drug penetration. In this context, the reduction of the metastatic potential of cancer cells on one hand and the re-education of a dysfunctional tumor microenvironment on the other could be beneficial for the treatment of pancreatic cancer as well as of other tumors sustained by a highly active stroma, such as breast cancer [121].
